# The Two-Way Association of Periodontal Infection with Systemic Disorders: An Overview

**DOI:** 10.1155/2015/793898

**Published:** 2015-08-03

**Authors:** Ravinder Nagpal, Yuichiro Yamashiro, Yuichi Izumi

**Affiliations:** ^1^Probiotics Research Laboratory, Graduate School of Medicine, Juntendo University, Tokyo 113-0033, Japan; ^2^Department of Periodontology, Graduate School of Medical and Dental Sciences, Tokyo Medical and Dental University, Tokyo 113-0034, Japan

## Abstract

Oral cavity that harbors diverse bacterial populations could also act as a site of origin for spread of pathogenic microorganisms to different body sites, particularly in immunocompromised hosts, patients, the elderly, or the underprivileged. A number of recent publications have advocated that patients with periodontal diseases are more susceptible to metabolic endotoxemia, inflammation, obesity, type 2 diabetes, and other related systemic complications, concluding that periodontal diseases could be a potential contributing risk factor for a wide array of clinically important systemic diseases. However, despite a significant increase in the prevalence of periodontal infections and systemic diseases in the past few decades, the fundamental biological mechanisms of connection between these ailments are still not fully explicated. Consequently, the mechanisms by which this bidirectional damage occurs are being explored with a concentric vision to develop strategies that could prevent or control the complications of these ailments. This paper attempts to summarize and hypothesize the diverse mechanisms that hint to a certain connection between the two prevalent chronic situations.

## 1. Introduction

**Figure 1:**
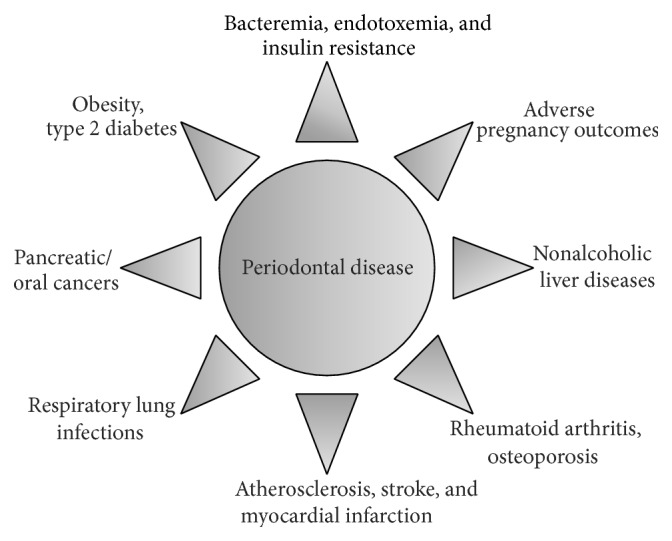
Diagram of periodontal disease leading to other complications.

Periodontitis is a multifactorial disease with numerous systemic or local risk factors playing a part in its clinical sequences. Periodontal diseases are influenced by various risk factors including ageing, smoking, oral hygiene, socioeconomic status, genetics, race, gender, psychosocial stress, osteopenia, osteoporosis, and other medical conditions including obesity and type 2 diabetes mellitus (T2DM) [1, 2], signifying that periodontitis does not occur merely as a consequence of plaque accretion but is also coupled with various host factors which could alter the consequence of the plaque on a particular individual. Recent findings have suggested that chronic low-grade inflammation is directly involved not only in the pathogenesis of obesity, diabetes, and their complications but also in the pathogenesis of periodontal diseases [3, 4], where cytokines play a central role in the host's responses to the periodontal biofilms. A number of diverse studies have indicated that periodontal diseases may also be associated with a wide array of systemic diseases and conditions (Figure 1). The primary putative facts that support the biological connection between periodontitis and systemic diseases are (a) usual implication of infection in the pathogenesis of both diseases, (b) transient and low-grade bacteremia and endotoxemia caused by periodontal diseases, (c) systemic immune responses and inflammation triggered by periodontal diseases, (d) expression of virulence factors by periodontal pathogens, and (e) presence of periodontal pathogens in nonoral tissues like atheromatous plaques [5–7]. Although the detailed mechanisms underlying this association are still unclear, available reports evidently demonstrate a bidirectional link between the mechanism of periodontal diseases and systemic/metabolic diseases where both conditions could aggravate each other [1, 8, 9].

## 2. Periodontitis and Obesity/Diabetes: The Two-Way Complication

**Figure 2:**
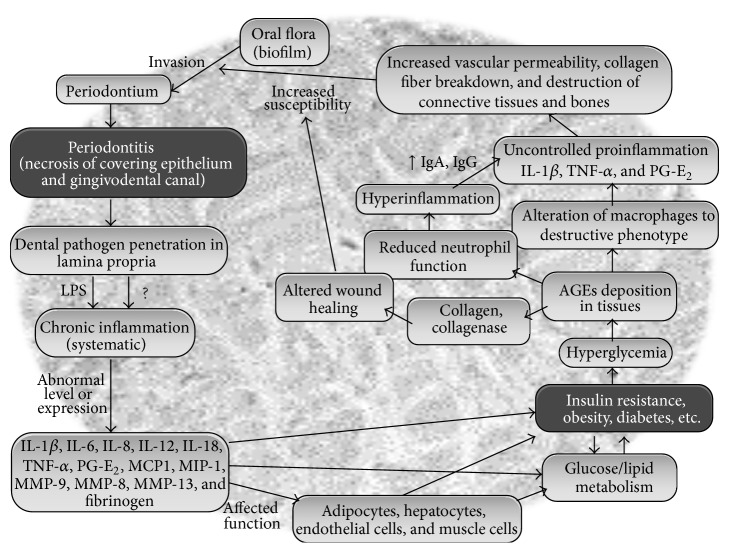
A summary of proposed connections between periodontal diseases and metabolic disorders such as obesity, insulin resistance, and type 2 diabetes (LPS: lipopolysaccharide; IL: interleukins; TNF: tumor necrosis factor; PGE2: prostaglandin E2; MCP: monocyte chemoattractant protein; MIP: macrophage inflammatory protein; MMP: matrix metalloproteinase; AGEs: advanced glycation end products; Ig: immunoglobulin).

Most of the mechanisms that support the influence of obesity and/or T2DM on periodontium generally share similar characteristics with those implicated in the typical complications of the diabetes [10]. For instance, in T2DM patients, hyperglycemia leads to a higher deposition of advanced glycation end products (AGEs) in tissues where these AGEs bind to the neutrophils and impair their normal functions. Further, these AGEs may also activate several unsought cell-surface receptors (RAGEs) which may alter the macrophages to a destructive phenotype. Both of these situations aggravate an uncontrolled production of proinflammatory cytokines and eventually lead to an increased vascular permeability, collagen fiber breakdown, and destruction of connective tissues and bones through increased lipid peroxidation and raised levels of IgA, IgG, and so forth, thereby making the diabetic patients more prone to periodontitis (Figure 2). Likewise, in patients with periodontal infections, the penetration of pathogen(s) (mainly* Porphyromonas gingivalis, Prevotella intermedia, Tannerella forsythia, Treponema denticola,* and* Aggregatibacter actinomycetemcomitans*) or their products in lamina propria may lead to endotoxemia and a state of systemic chronic inflammation through the leakage of endotoxins such as lipopolysaccharides (LPS) into the serum. This hyperinflammation may further affect the expression and functioning of important immunoinflammatory molecules such as IL-1*β*, IL-6, TNF-*α*, PGE2, IL-8, IL-12, and IL-18, thereby contributing to insulin resistance and an altered lipid and glucose metabolism [11]. Eventually, the functioning of various tissues and cells such as adipocytes, hepatocytes, and endothelial and muscle cells may get impaired, thereby leading to more chronic metabolic states, that is, obesity, T2DM, and so forth in these periodontitis patients (Figure 2).

Since periodontal diseases are infectious diseases, earlier studies emphasized primarily the possible variations in subgingival microflora of patients with and without T2DM. However, the findings of altered functions of neutrophils, monocytes, and macrophages in people with T2DM gradually shifted the research focus towards possible discrepancies in the immunoinflammatory responses between people with and without T2DM [10]. Impaired adherence, chemotaxis, and phagocytosis capacities of neutrophils (the first line of host defense) may avoid the destruction of bacteria in the periodontal cavity and lead to an increased periodontal damage. Since altered wound healing is another frequent problem suffered by people with T2DM, distorted periodontal wound healing responses to persistent microbial encounters in patients with persistent hyperglycemia may also contribute to an increased bone and attachment loss. Given that the inflammatory cells such as monocytes and macrophages harbor receptors for AGEs, the accumulation of AGEs in T2DM patients may also intensify the proinflammatory responses to periodontal pathogens. Further, the interactions between AGEs and their receptors on inflammatory cells could stimulate hyperproduction of proinflammatory cytokines such as IL-1*β* and TNF-*α*, consequently increasing the risk or occurrence of periodontal diseases in T2DM patients (Figure 2).

Patients with inflammatory periodontal diseases usually have higher serum levels of proinflammatory cytokines [12]. Since the hyperinflammatory immune cells could intensify the production of proinflammatory cytokines in T2DM patients, this could also increase the insulin resistance and complicate the control of diabetes. Nonetheless, hyperglycemia and AGEs are only a few of the numerous potential factors that are implicated in the complications of obesity/T2DM as well as in the pathophysiology of periodontitis in people with the diabetes [4]. Polymorphonuclear leukocytes and alterations in the collagen metabolism could be another possible reason for higher predisposition of T2DM patients towards periodontal diseases. The formation of AGEs may influence the collagen stability and vascular integrity and could also aggregate macrophage and monocyte receptors, thereby aggravating the susceptibility to periodontitis through the stimulation of IL-1 and TNF-*α* [13] (Figure 2). These inflammatory cytokines are known to stimulate the insulin resistance and several other chronic inflammatory complications including periodontitis [14]. Moreover, the fact that TNF-*α* and IL-6 are produced in the adipose tissues could also support the shared link between obesity, T2DM, and periodontitis [15].

## 3. Periodontitis and Obesity/Diabetes: Underlying Mechanisms

The principal mechanisms that link oral infection with systemic diseases are (a) metastatic spread of infection from the oral cavity as a consequence of transient bacteremia, (b) metastatic spread of cellular injuries because of the circulation of oral bacterial toxins, and (c) metastatic spread of inflammation through the immunological injuries triggered by oral bacteria [16]. The association between periodontal diseases and systemic inflammation is also supported by the observation that the chronic inflammation is a significant factor in the fundamental pathophysiology of both of these ailments and that the local/systemic variations triggered by periodontitis may also lead to a chronic inflammatory state that can increase the susceptibility to metabolic syndromes [11] (Figure 2).

Given that the adipose tissue, particularly the white adipose tissue (WAT), acts as a main endocrine organ secreting a number of bioactive substances such as adipocytokines, TNF-*α*, leptin, adiponectin, and resistin, it can also affect the periodontal response or can also be affected during periodontal infections [12]. For instance, a negative correlation of the degree of periodontal damage with leptin concentration in gingival crevicular fluid of periodontitis patients and a positive correlation with leptin concentration in the serum indicate a negative correlation of gingival crevicular leptin concentration and a positive correlation of serum leptin during the progression of clinical attachment level [17]. Since the gingival inflammation could also cause vasodilatation, it may also increase the serum levels of leptin which would further act as a defense mechanism of the body to battle the periodontal inflammation [18]. The serum levels of resistin have also been observed to be elevated in people with periodontitis, indicating that it may also play a role in periodontitis [19]. It has been observed that in patients with periodontitis and T2DM, effective glycemic control may improve bleeding on probe lesions by improving the inflammation at gingival sites of periodontal tissues [20], while the treatment of periodontitis with topical antibiotics may ameliorate the periodontal status and glycemic control with an elevation of serum adiponectin and reduced HbA1c [21].

Because of the predominated role of gram-negative anaerobic bacteria in periodontal infections, the ulcerated pocket epithelium turns into a chronic source of systemic challenge from bacteria, bacterial products, and locally produced inflammatory mediators. Further, as a consequence of the high vascularity, the inflamed periodontium may act as an endocrine-like source of inflammatory mediators (such as TNF-*α*, IL-6, and IL-1) which are significant in periodontal inflammation and may also influence glucose and lipid metabolism [22] (Figure 2). In view of the fact that osteoblasts, which are involved in bone turnover, also express Toll-like receptors- (TLRs-) 1, 4, 5, 6, and 9, while osteoclasts express TLRs-1, 2, 3, 4, 5, 6, 7, 8, and 9 [23, 24], it is also likely that the TLRs signaling within the alveolar bone may cause an inflammatory response to invading pathogens, and this initiation of a cascade of proinflammatory cytokines within the alveolar bone could lead to a pathological resorption of bone through excessive or extended production of osteolytic host molecules such as IL-1, TNF-*α*, and prostaglandin E2 (PGE2) which may further stimulate the osteoblast inhibition and osteoclast activation through the receptor activator of nuclear factor kappa-*β* (NFk-*β*) ligand.

Since the serum levels of total cholesterol, low-density lipoprotein cholesterol, and triglycerides have been observed to be higher in periodontal patients, periodontitis may also be a risk factor for hyperlipidemia [25]. The hyperactivity of white blood cells which is caused by the hyperlipidemia may also increase the production of oxygen radicals that are often linked with the development of periodontitis, and this decline in the antioxidant ability in periodontitis patients could also trigger the development of insulin resistance [18]. Such variations in the phenotype of immune cells due to the elevated levels of lipids and serum proinflammatory cytokines in chronic periodontitis may also support the two-way correlation between the two diseases [26] (Figure 2). However, it still remains to be fully revealed if (and how) periodontitis provokes the higher lipid levels or higher lipid levels influence the periodontitis.

## 4. Periodontal Infections and Other Systemic Diseases

There has been a significant interest in the possible association between oral and systemic diseases in the past few decades [27–29], especially after the case-control study by Mattila et al. [30] who noticed a significant association between poor dental health and acute myocardial infarction in the patients, as compared to control subjects. Subsequently, various epidemiological studies have investigated and supported a causal association of periodontitis with several clinical systemic diseases, including cardiovascular disease [31, 32], diabetes [33], respiratory disease [34], adverse pregnancy outcomes [35], Alzheimer's disease [36], pancreatic cancer [37], and cerebral infarction [38]. In addition to the chronic inflammation triggered in response to the oral pathogens, periodontal infection may also result in tooth loss, oral pain, poor mastication, and several nutritional defects and may also be expected to be related with Alzheimer's disease and dementia [39–41]. Decreased mastication due to oral pain and tooth loss could also result in reduced acetylcholine synthesis which may cause several learning and memory problems [42]. In addition to the incidences of hypertension and diabetes mellitus, the number of lost teeth has also been found to be higher in patients with silent infarctions and cerebral white matter changes, as compared to healthy group, thereby hinting that periodontal infections may also be a predictor of stroke and cognitive impairment [43].

**Figure 3:**
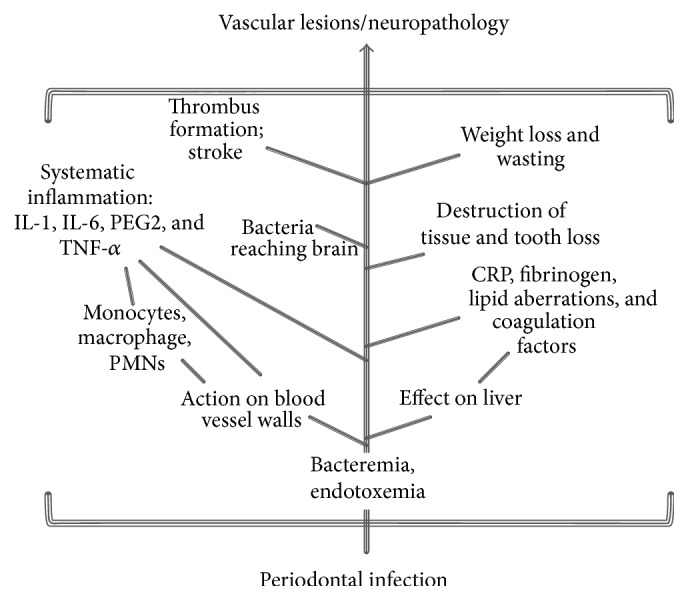
Potential consequences of periodontal disease leading to stroke, infarction, atherosclerosis, and other neuropathological complications.

Although the precise role and underlying mechanisms of periodontal infections in the pathology of systemic diseases still remain to be completely established, several hypotheses have been proposed based on the findings of various clinical and epidemiological investigations (Figure 3) [5, 44–46]. The primary factor includes the shared risk factors among oral infection and systemic diseases, such as genetic or environmental factors including age, smoking, lifestyle, and socioeconomic status. Another mechanism is the systemic inflammation against the local infection or circulating bacteria and associated higher levels of circulating inflammatory biomarkers which could play a contributing role in systemic disease. Also, the significant role of infection and inflammation in diseases such as atherosclerosis, cardiovascular disease (CVD), and coronary heart disease (CHD) also underscores the possible etiological role of periodontal infections in these diseases [28, 30, 47–50]. The pathogens from periodontal pockets may also enter into the connective tissues, endothelial cells, and the bloodstream and thus could lead to the formation of thrombus by platelet aggregation degrading collagen [51–53]. Chronic periodontal infections can contribute to atherogenesis either directly by triggering the platelet aggregation and invasion causing damage to endothelial cells or indirectly by stimulating the synthesis of intracellular adhesion molecules and production of antibodies against bacterial LPS thereby causing a discrepancy of the immune system [54, 55]. Moreover,* P. gingivalis *and* A. actinomycetemcomitans* have also been detected in atheromatous plaques of CVD patients, indicating a connection between periodontal infections and the formation of atherogenic lesions [56–59]. A recent systematic meta-analysis of epidemiologic literature has also suggested that periodontal infection could be an independent risk factor for CHD (although relatively weak) and that various measures of periodontal infections could explicate 30% increase in risk of CHD [60].

### 4.1. Periodontitis and Fatty Liver

In addition to insulin resistance, obesity, diabetes, and oxidative stress, periodontal diseases may also be implicated in the pathogenesis of nonalcoholic fatty liver disease (NAFLD) and nonalcoholic steatohepatitis (NASH). Since periodontal pathogens, their endotoxins, and/or cytokines released from the organisms could invade into the blood circulation and cause bacteremia, endotoxemia, and inflammation, such periodontal infections may also be implicated as an independent risk factor for NAFLD/NASH. For instance, the incidences of* P. gingivalis* infection have been found to be significantly higher in NAFLD patients as compared to healthy subjects [61], hinting at the involvement of* P. gingivalis* infection in the onset of NAFLD. Further, observation of a lower serum albumin levels in* P. gingivalis*-positive NASH/NAFLD patients indicates that* P. gingivalis* infection may lead to a decreased liver function thereby progressing to the pathogenesis for NAFL or NASH. Interestingly, periodontal treatment has been found to improve the liver functional parameters such as serum aspartate aminotransferase and alanine aminotransaminase in NAFLD patients, again signifying the fact that* P. gingivalis*-positive periodontitis may be a risk factor for the progression of NAFLD. Since* P. gingivalis* virulence strains could release LPS and TNF-*α*, its infection may lead to the inflammation of other systemic organs, besides the local gingiva.* P. gingivalis* may also enter into the blood circulation from the gingiva after widespread periodontal processes such as chewing, tooth-brushing, subgingival irrigation, and dental extractions again supporting the hypotheses that* P. gingivalis* or other similar periodontal infections may also be an infrequent risk factor for the progression of NAFLD or NASH [62].

### 4.2. Periodontal Disease and Respiratory Infections

**Figure 4:**
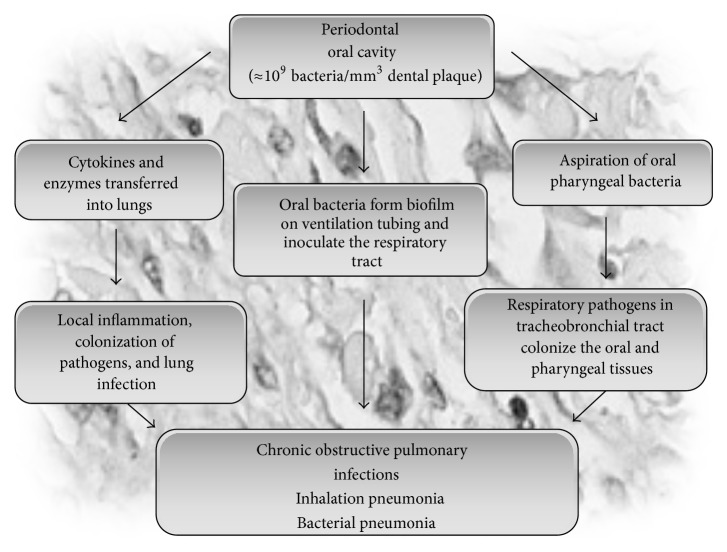
Possible role of periodontal infection in respiratory diseases.

Poor oral health may also predispose the host to respiratory diseases, particularly in high-risk patients such as residential nursing patients, hospitalized patients, elderly, smokers, and the underprivileged. In periodontal infections, the aspiration or hematogenous spread of bacteria from the oropharynx into the lower respiratory tract and the consequent infection of respiratory ducts can easily cause respiratory infections such as pneumonia and chronic obstructive pulmonary diseases [63, 64] (Figure 4). Since the oral cavity is adjacent to the trachea, it could be an easy entrance for the immigration and colonization of respiratory pathogen. Respiratory pathogens may infrequently populate dental plaques and may also be aspirated/inhaled from the oropharynx into the upper airway and then the lower airway where they may adhere to the alveolar and bronchial epithelium [65–67]. In periodontal patients, one mm^3^ of dental plaque may contain about 10^9^ bacteria and hence could serve as a persistent pool for potential oral/respiratory pathogens which could be shed into the saliva and aspirated into the lower respiratory tract and the lungs to cause infection [68] (Figure 4). Further, the cytokines and enzymes induced from the inflamed periodontal tissues may also relocate into the lungs and trigger local inflammatory processes and lung infections [34]. Also, in periodontal diseases, poor oral hygiene may result in a higher concentration of oral pathogens in the saliva, and these pathogens may be aspirated into the lung overcoming the immune defenses and assist the pulmonary pathogens in inhabiting the upper airways. Generally, in healthy scenarios, the respiratory tract is capable of defending against aspirated bacteria. However, in periodontal diseases, the disturbed oral hygiene, reduced salivary flow, decreased cough reflex, dysphagia, and other disabilities can predispose the patients to a high risk for pulmonary infections [69–74].

### 4.3. Periodontal Diseases and Cancer(s)

A number of clinical and epidemiological studies have observed higher risks of oral, gastrointestinal, lung, and pancreatic cancers in subjects with periodontal disease, thereby linking oral bacteria with the etiology of these cancers. In addition to tobacco and alcohol consumption, a poor oral hygiene could also be a possible risk factor for oral cancers [75, 76]. Several case-control studies have found tooth loss to be associated with a higher oral cancer risk [77, 78], indicating that tooth loss may contribute to oral cancers either by promoting the initiated tumors or by some other complex mechanism(s). Several reports have also suggested that oral bacteria could contribute to the cancers of upper gastrointestinal tract including aerodigestive tract, esophagus, and stomach, possibly through the similar inflammatory mechanisms as that of* Helicobacter pylori *[79–82]. However, available evidences are inadequate, and hence further studies are awaited to validate a definite association between periodontal diseases and gastrointestinal cancers.

### 4.4. Periodontal Diseases and Adverse Pregnancy Outcomes

Approximately half of the perinatal deaths or congenital neurological deficits are caused as a result of premature births [83]. Incidences of intrauterine infection and inflammation are known to be a significant contributor to majority of the preterm deliveries [83]. Several studies have speculated that periodontal diseases (besides appendicitis, pneumonia, or other remote infections) may also trigger preterm labor, prematurity, and low birth-weight, primarily through (a) the possible hematogenous invasion of oral pathogens and/or their metabolites/toxins, (b) circulation of the inflammation by-products through the bloodstream, and (c) subsequent maternal/fetal immune responses against the invading pathogens, toxins, inflammatory inducers, and so forth [84–89]. Several clinical and observational investigations, however, have failed to observe any significant association between periodontal disease and the occurrence of preterm births or low-birth weight, and hence more investigations are requisite to resolve this paradox [90–93].

## 5. Periodontal Diseases and Overall Health:* The Nonclinical Links*

In addition to the various clinical, immunological, or molecular mechanisms that link periodontal infection with the systemic health, periodontal diseases may also have an indirect effect on the overall health status of the patient which could further exaggerate the health complications. Since periodontal disease leads to oral pain and teeth loss, it may result in poor mastication, less appetite, and less food intake which can cause nutritional deprivations. The oral pain may also cause sleep deprivation, thereby causing an upset behavior and hypertension. Bad breath and oral pain may also negatively affect the social routine of the patient and reduce the social and physical activities of the patient. The high cost of treatment regimen may also disturb the socioeconomic status of the patient. All these factors such as oral pain, teeth loss, bad breath, deprived nutrition and sleep, reduced physical and social activities, and depression may altogether make the patient more vulnerable to low self-esteem, hypersensitivity, and weakened immune system and hence may adversely affect the overall health.

## 6. Concluding Remarks

Although the recent evidences have supported the role of periodontal infection and consequent inflammation in diseases such as obesity, type 2 diabetes, cardiovascular disease, and gastrointestinal and pancreatic cancers, the precise etiological role of periodontal infections still needs to be deciphered completely. Yet, the available literature is sufficient to establish that the periodontal diseases may be a significant risk factor for various systemic disorders, and hence future studies are anticipated to elucidate the mechanisms through which the periodontal diseases and systemic diseases affect each other. Nevertheless, it is only after the precise understanding of these diseases that the attention could be shifted from the treatment of these ailments to their prevention for a healthier socioclinical scenario.
